# Raspberry Ketone Accumulation in *Nicotiana benthamiana* and *Saccharomyces cerevisiae* by Expression of Fused Pathway Genes

**DOI:** 10.1021/acs.jafc.3c02097

**Published:** 2023-09-01

**Authors:** Markus Laurel, Dominik Mojzita, Tuulikki Seppänen-Laakso, Kirsi-Marja Oksman-Caldentey, Heiko Rischer

**Affiliations:** VTT Technical Research Centre of Finland Ltd., P.O. Box 1000, FI-02044 Espoo, Finland

**Keywords:** raspberry ketone, transient expression, gene fusion, yeast, tobacco, flavor, metabolic engineering

## Abstract

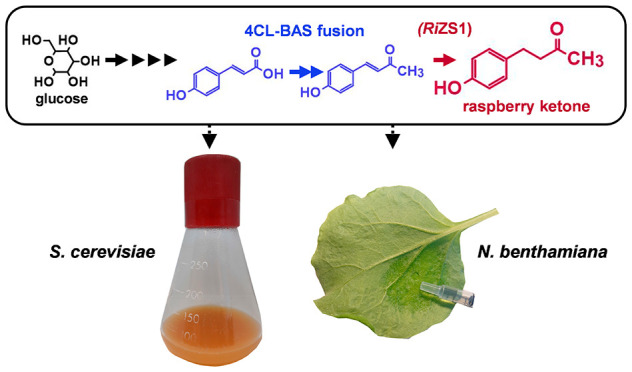

Raspberry ketone has generated interest in recent years both as a flavor agent and as a health promoting supplement. Raspberry ketone can be synthesized chemically, but the value of a natural nonsynthetic product is among the most valuable flavor compounds on the market. Coumaroyl-coenzyme A (CoA) is the direct precursor for raspberry ketone but also an essential precursor for flavonoid and lignin biosynthesis in plants and therefore highly regulated. The synthetic fusion of 4-coumaric acid ligase (4CL) and benzalacetone synthase (BAS) enables the channeling of coumaroyl-CoA from the ligase to the synthase, proving to be a powerful tool in the production of raspberry ketone in both *N. benthamiana* and *S. cerevisiae*. To the best of our knowledge, the key pathway genes for raspberry ketone formation are transiently expressed in *N. benthamiana* for the first time in this study, producing over 30 μg/g of the compound. Our raspberry ketone producing yeast strains yielded up to 60 mg/L, which is the highest ever reported in yeast.

## Introduction

Flavors and fragrances are an integral part of everyday life. Recently, due to public awareness, there has been an increase in the demand for sustainably produced flavor compounds derived from natural sources.^1^ Yeast can be used as a platform for production and extraction of raspberry ketone as a flavor compound but also for adding flavor to fermented beverages.^2^ The use of genetically modified yeasts might also become a viable option in the future in Europe. Since the approval of the first genetically modified (GM) yeast for food production in USA and Canada two decades ago,^3^ GM yeast have been used in enhancing the flavor profile of beer.

Raspberry ketone, 4-(4-hydroxyphenyl) butan-2-one, is the major aroma component in raspberry fruits (*Rubus idaeus*)^4^ at a concentration of 1–4 mg/kg.^5^ It is also found in various other berries, vegetables, and plants, in minute quantities, making industrial scale extraction economically nonviable. There is a high demand for the compound for food and beverage industries as a flavor agent^6^ with a low odor threshold of 1–10 ppb^4,7^ and a market value up to 6–10 million euros.^8^ Natural raspberry ketone is the second most expensive flavor compound only behind natural vanillin, demanding prices of 3000–20000 €/kg.^9^ There is also a growing interest for the compound as a weight reduction supplement,^10^ a skin lightening agent,^11^ and hair growth promoter in alopecia patients.^12^ Raspberry ketone can be produced chemically utilizing petrochemicals and other toxic reagents; however, the growing interest for all-natural compounds has made bioengineering a viable approach to produce the compound in an environmentally sustainable way.

**Figure 1:**
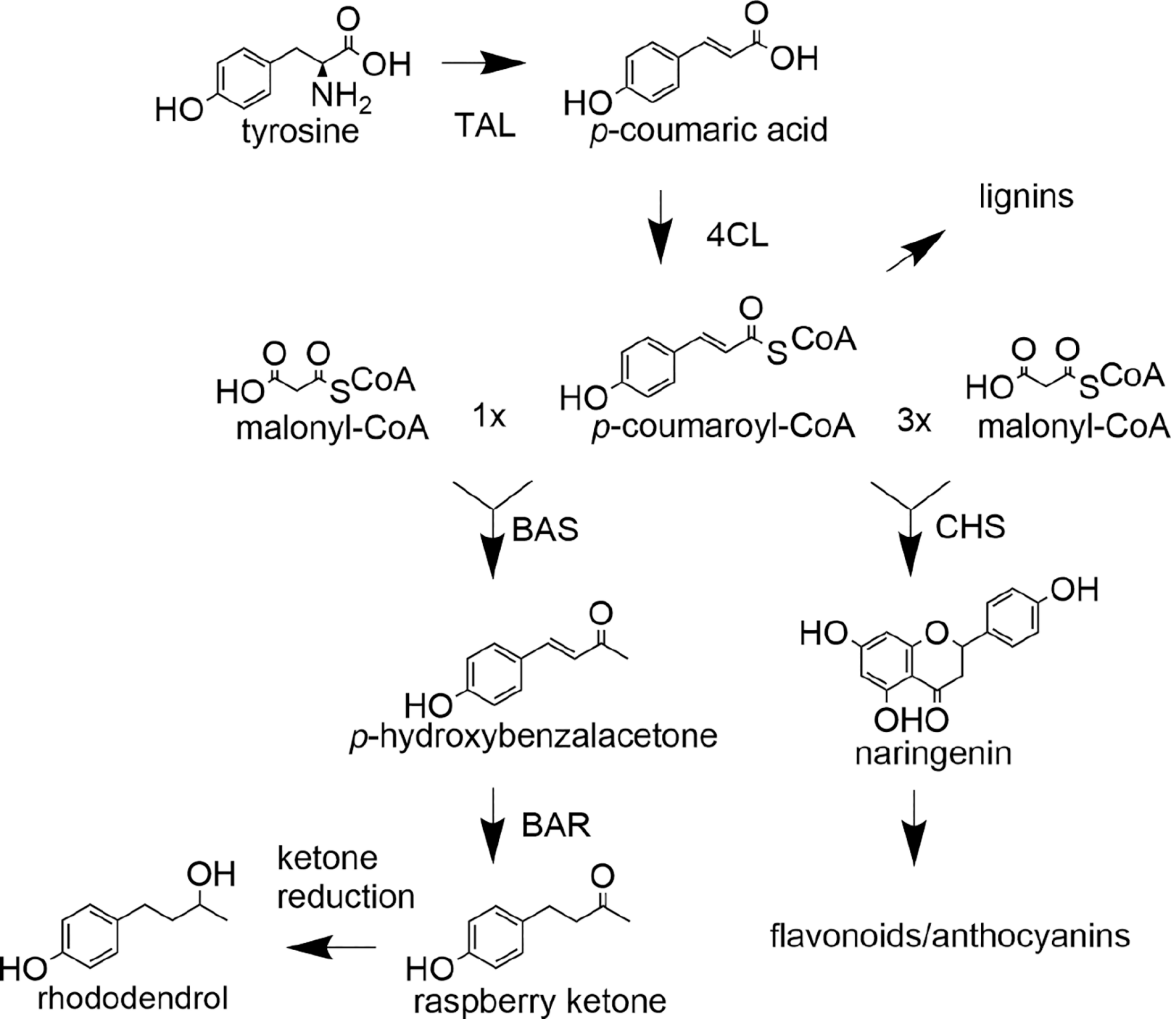
Enzymes involved in the biosynthesis of raspberry ketone and the utilization of *p*-coumaroyl-CoA in other pathways: tyrosine ammonium lyase (TAL), 4-coumarate:CoA ligase (4CL), benzalacetone synthase (BAS), benzalacetone reductase (BAR), and chalcone synthase (CHS).

The biosynthetic pathway leading to the production of raspberry ketone via the phenylpropanoid pathway is known from *R. idaeus*,^13−16^ and the enzymes involved have been characterized (Figure 1). Phenylalanine is deaminated by phenylalanine ammonium lyase (PAL), yielding *trans*-cinnamic acid,^17^ which is then hydroxylated by a cytochrome P450-dependent monooxygenase, cinnamate 4-hydroxylase (C4H) to yield 4-coumarate.^18,19^ An ATP-dependent reaction catalyzed by 4-coumarate:coenzyme A (CoA) ligase (4CL) leads to *p*-coumaroyl CoA.^20^ Benzalacetone synthase (BAS),^21,22^ an enzyme belonging to the chalcone synthase superfamily of type III polyketide synthases (PKS), condenses one molecule of *p*-coumaroyl-CoA with one molecule of malonyl-CoA to produce a diketide benzalacetone scaffold: 4-hydroxybenzalacetone. The latter is finally reduced by a NADPH-dependent reductase (BAR) in this case *Ri*ZS1^1,14^ to produce raspberry ketone.

The phenylpropanoid pathway is crucial for plants to synthesize a broad variety of critically important metabolites. The pathway is tightly regulated and is postulated to involve supramolecular complexes, or metabolons, channeling the flux of metabolites.^23,24^ It would therefore not be a surprise that intermediates in the pathway such as *p*-coumaroyl-CoA are not abundantly available for artificially expressed enzymes, leading to deviating pathways. The engineered production of compounds derived from the phenylpropanoid pathway, such as resveratrol, is greatly enhanced by utilizing a synthetic fusion sequence of 4CL and the corresponding synthase, as previously shown to function in a plant system,^25^ leading to the accumulation of resveratrol in *Nicotiana tabacum*.

Microbial hosts have been extensively used in studies for heterologous production of raspberry ketone;^9,26,27^ however, to the best of our knowledge, there have only been a few studies utilizing plants as an expression host. Bioconversion has been used by Häkkinen et al. as a route to convert betuligenol and 4-hydroxybenzalacetone to raspberry ketone utilizing hairy root cultures and plant cell cultures, producing up to 29 μg/g (dry weight) in *Catharanthus* hairy root cultures.^28^ However, this procedure requires time-consuming laboratory setups. Stable transformation of *N. tabacum* with multigene expression and upregulation of the phenylpropanoid pathway with PAP1 transcription factor was utilized by Koeduka et al. to produce up to 2.2 μg/g (fresh weight) of raspberry ketone glucoside, but no aglycone was produced in tobacco leaves.^44^ In general, however, plants have been extensively utilized for producing a broad array of compounds ranging from alkaloids to polyphenols and terpenes, mostly via stable transformation.^29^

In this research, we produced raspberry ketone by building an artificial biosynthetic pathway, utilizing a synthetic fusion-enzyme (4CL-BAS) combined with a highly efficient synthetic expression system (SES)^30^ to transiently express the genes in *N. benthamiana* and constitutively in yeast. Furthermore, we increased the production of raspberry ketone in yeast by introducing tyrosine feedback insensitive (*aro*7^G141S^)^31^ and (*aro*4^K229L^)^32^ to enhance the availability of tyrosine, the precursor of *p*-coumaric acid, for tyrosine ammonium lyase (TAL), in *de novo* raspberry producing strains. Although it has been illustrated in *in vitro* enzyme studies that a serine to valine mutation in the amino acid sequence of *Rp*BAS at position 331 increases benzalacetone-forming activity 2-fold compared to that in the natural *Rp*BAS,^22,33^ we show that this does not correspond to *in vivo* expression of the gene. In our experiments, natural BAS outperforms the mutated enzyme in the formation of raspberry ketone in both tobacco and yeast.

## Materials and Methods

### Media and Culture Conditions

*Escherichia coli* cultures were grown in LB culture medium (10 g/L tryptone, 5 g/L yeast extract, and 10 g/L NaCl)^34^ supplemented either with 100 mg/L spectinomycin or 100 mg/L ampicillin at 200 rpm and 37 °C. *Agrobacterium tumefaciens* EHA-105 cultures were similarly grown in LB medium supplemented with 100 mg/L spectinomycin and 15 mg/L rifampicin at 200 rpm and 28 °C. *S. cerevisiae* cultures were grown in YPD medium (10 g/L yeast extract, 20 g/L peptone, and 20 g/L d-glucose) in 50 mL of liquid medium in 250 mL Erlenmeyer flasks at 220 rpm. SCD-HIS plates (histidine deficient synthetic complete medium supplemented with 20 g/L d-glucose) were used for selecting the transformant of yeast strains. Cultures were supplemented with and without the precursor *p*-coumaric acid (*p*-HCA) (Merck, Rahway, USA) in doses of 3.9 mg (in 80 μL of absolute ethanol) added every day to a total of 27 mg during the seven-day incubation. Yeast strains with full pathways were grown in YPD for 7 days in culture conditions of 30 °C and at 220 rpm.

### Enzymes and Reagents

FastDigest restriction endonucleases were purchased from Thermo Fischer (Bellefonte, USA) and were used for all of the reactions in this experiment. Phusion polymerase from Thermo Fisher was utilized for generating PCR fragments, and for extracting PCR fragments, a NEB Monarch (New England Biolabs, Ipswich, USA) gel purification kit was used for extracting PCR fragments. A NEBuilder HiFi kit (New England Biolabs, Ipswich, USA) was used for Gibson cloning, and for plasmid extraction from *E. coli*, a GeneJet Plasmid Miniprep Kit from Thermo Fischer was used. DreamTaq polymerase from Thermo Fischer was used for colony PCR.

### Plasmid Construction for Transient Expression in *N. benthamiana*

For transient expression experiments, *N. benthamiana* codon-optimized synthetic genes were ordered from GeneArt (Thermo Fischer, Bellefonte, USA) and Genscript (Genscript Biotech, Piscataway, Table S1). The Gateway based plasmid pK2GW7_*Nb*-SES_mCherry incorporating a SES promoter^35^ was digested with *Pac*I and *BamH*I, and a vector backbone was purified with gel electrophoresis. Synthetic *At*4CL1 gene fragment was designed to have a 30 bp Gibson assembly (GA) flank upstream of the sequence, including a *Pac*I restriction enzyme site and lacking a termination codon, followed by the linker sequence GGATCTGGC (GSG). The sequence for the mutated *Rp*BAS(S331V) gene was designed to have a 30 bp upstream GA flank homologous to the *At*4CL sequence and 30 bp downstream flank with a *BamH*I restriction enzyme site. The fragments were assembled with the GA method according to manufacturer protocol, resulting in the vector p_Nb4CL-BAS_M. The gene fragment for *Ri*ZS1 was designed to contain GA flanks and *Pac*1 and *BamH*I restriction sites and was assembled according to GA protocol, resulting in p_Nb-ZS1. The gene constructs for natural *Rp*BAS and *Rp*BAS linked to *At*4CL1 were made by designing GA primers and were generated by PCR from pUC57 BAS plasmid from Genscript. The fragments were purified and assembled according to GA protocol resulting in the vectors p_Nb4CL-BAS and p_NbBAS. The construct for *At*4CL was made by designing GA primers and performing a PCR on p_Nb4CL-BAS after which the fragment was purified and assembled according to GA protocol generating p_Nb4CL. Empty vector was generated from pK2GW7_*Nb*-SES_mCherry by digesting with *Bam*HI and PacI and cloning empty GA flanks to the vector backbone. *E. coli* strain Top10 was used for cloning all plasmids. Colony PCR was used to identify the correct constructs, after which plasmids were sent for sequencing (Microsynth Seqlab, Göttingen, Germany).

### Plasmid Construction for *S. cerevisiae*

Plasmids cloned for *S. cerevisiae* expression experiments were based on pRSET and pLL plasmids. Synthetic *S. cerevisiae* codon optimized gene fragments were synthesized by GeneArt (Thermo Fischer, Bellefonte, USA). The bidirectional plasmid pR_ScZS1_4CL-BAS_M was made by digesting the plasmid B10598 from the VTT plasmid collection with *Pac*I restriction enzyme to extract the SES promoter region. The same plasmid was digested with *Xho*I and *Sal*I to generate the vector backbone. The fragments Pc4CL-GSG and *Rp*BAS(331V) were amplified from the B10598 plasmid, containing the genes Pc4CL and *Rp*BAS(S331V), with GA primers containing overlapping flanks and the sequence for substituting one of the *Pac*I sites with *Swa*I. The fragments were assembled according to the GA protocol with the vector backbone and promoter region. The plasmid pR_ScZS1_4CL-BAS was generated by assembling the vector, promoter, and Pc4CL-GSG fragments from the previous cloning, and a *S. cerevisiae* codon optimized synthetic gene fragment *Rp*BAS according to the GA protocol. This vector preserves the double *Pac*I restriction sites around the promoter region due to a slightly different design. The CRISPR plasmid for expressing *Fj*TAL and Aro7^G141S^ was created by digesting pLL-038 plasmid from VTT plasmid collection, with restriction enzymes *BamH*I and *Sal*I, to extract the vector, and the previously made pR_ScZS1_4CL-BAS_M with *Pac*I and *Swa*I for extracting the Bidirectional SES promoter. Synthetic gene fragments were assembled with the vector and promoter region, with GA producing the plasmid pL_TAL_ARO7. *E. coli* strain Top10 was used for cloning all plasmids. Colony PCR was used to identify correct constructs, after which plasmids were sent for sequencing (Microsynth Seqlab, Göttingen, Germany). Table 1 lists all the primers that were used throughout our study.

**Table 1:** List of Primers

*Nicotiana benthamiana*	
Nb_BAS_GA_F	CATAAATCTTCTCAGATCTCTTCCAATTTTCTTTAATTAAAATGGCAACTGAGGAGATGAAGAAATTGG
Nb_BAS_GA_R	CAATAATGATGTAAGAAAGATATATAGCTATTAGGATCCCTAGCTAATTACGGGCACACTGCGTA
Nb_BAS-l-GA_F	ATGGCAACTGAGGAGATGAAG
Nb_4CL_GA_F	CTTCATAAATCTTCTCAGATCTCTTCC
Nb_4CL_GA_R	CAATAATGATGTAAGAAAGATATATAGCTATTAGGATCCAATCAATCCATTTGCTAGTTTTGCC
Nb_MT2Bcp_F	CTTCTTGACCTTGACCCTTCC
Nb_BAS_qPCR_F	TTCAGATGGCCCACGAAA
Nb_4CL-qPCR_F	CGGAGACGGAGAAGTATGATTTG
Nb_AT2G-ter_R	GCTGCAACATTGAAACGAATACAC

*Saccharomyces cerevisiae*	
Sc_RiZS1_GA_F	CCTACACTCTACATATCCACACCAATCTACTACAATTAATTTAAATAATGGCATCTGGCGGAGAAATGC
Sc_RiZS1_GA_R	CACAAACGTTGAATCATGAGTTTTATGTTAATTAGCGTCGACTCACTCTCTGGAAACAACCACCACC
Sc_4-CL-l_GA_R	CAGTAGCCAATTTCTTCATCTCTTCGGTAGCCATGCCAGATCCTTTTGGCAAGTCACCAGATGCAATTTTAGC
Sc_4CL-l_GA_F	TAATTGTAGTAGATTGGTGTGGATATGTAGAGTGTAGG
Sc_4CL-l-S331V_GA_R	GCCATAACTGTTGCCAATTTCTTCATTTCTTCTGTAGCCATGCCAGATCCTTTTGGCAAGTCACCAGATGCAATTTTAGC
Sc_BAS-qPCR_F	CCATTTGGGTTGTTACGCTG
Sc_GRE3t_R	GGAGACCACAACGGTTTCCCTC
Sc_114cP_F	GAACCCCTCGTTCTGTCTTACCTTC
Sc_4CL-qPCR_R	CCTTGACCTAACTTAGCGTTTG
Sc_ARO7_F	CTGATGAAACTCCCTTCTTTCCT
Sc_ARO7_R	ATCCTTCTTTCACCTGACTCGT
Sc_FjTAL_F	TTCAGGTGTTCACCCATCCG
Sc_FjTAL_R	AGCGCAGGAAGATTTCAAGGA

### Transient Expression in Tobacco

Wild type *N. benthamiana* plants were grown in the greenhouse under a 16/8 photoperiod at 25 ± 1 °C for 4–6 weeks before agroinfiltration. Transformed *A. tumefaciens* cultures were grown in liquid LB-media overnight. The optical density at 600 nm was adjusted to 0.8 with an infiltration buffer (10 mM MES, 10 mM MgCl_2_, and 200 μM acetosyringone). *Agrobacterium* cultures were then incubated for 1 h prior to infiltration at room temperature with gentle mixing at 70 rpm. Before infiltration, the *Agrobacterium* cultures were mixed in a ratio of 1:1. The leaves of three *N. benthamiana* plants were infiltrated on the adaxial side by either individual constructs or combinations using a needleless syringe.

### Yeast Transformation and Genome Editing

Yeast strain H4590 (Table 2), based on CEN.PK102-5B (VTT culture collection), incorporating SES synthetic transcription factor cassette integration using SES for high constitutive gene expression, was used as a base for all the transformations for raspberry ketone producing strains. Pc4CL fusion to *Rp*BAS was constructed to validate the function of a synthetic fusion in the production of raspberry ketone in *S. cerevisiae*. The bidirectional sequence for *Ri*ZS1 and 4CL-BAS(S331V) was integrated, after digestion with restriction enzyme NotI, into the GRE3 locus of the yeast strain H6709 needing external *p*-HCA feeding by using homologous recombination using the protocol by Gietz and Schiestl.^36^ Yeast strain H6708, utilizing a natural BAS sequence instead of BAS(S331V), was made similarly. Yeast strain H5304, which is a progeny of H4590, with an addition of a tyrosine feedback insensitive mutation of Aro4 was used as a base for *de novo* raspberry producing strains. Malonyl CoA production was upregulated by integrating an Snf1p-dependent acetyl CoA-carboxylase (ACC1) with mutated phosphorylation sites (S659A, S1157A) from *S. cerevisiae* and a codon optimized 4-phosphopantetheinyl transferase (NpgA) from *Aspergillus nidulans* using a bidirectional SES plasmid B13474 (VTT plasmid collection) into the LEU locus of the previously made strain producing a *de novo* coumaric acid producing yeast strain. Genomic integration was mediated with CRISPR-Cas9, based on a 2 μ multicopy Cas9-gRNA plasmid with a nourseothricin *N*-acetyl transferase gene (NAT) for nourseothricin selection. Donor DNA was expressed in an integrative bidirectional eight binding site SES plasmid with outward orientated synthetic core promoters expressing Aro7 and *Fj*TAL. Plasmid was cut with MssI FastDigest restriction enzyme at the X-1 integration flanks prior to transformation. The transformation was performed using the protocol by Gietz and Schiestl^36^ using 0.5 μg of Cas9-sgRNA plasmid and 2.5 μg of donor DNA, with an addition of a 3 h incubation in YPD medium before plating the transformed H5304 strain onto +NAT selection medium. Correct transformants were selected by PCR for Aro7 and FjTAL, and Cas9-gRNA plasmid was lost by growing yeast on YPD plates and choosing colonies that do not grow on +NAT selection, producing strain H6711. PCR was performed on selected yeast strains to confirm transformation (Table 1). Thereafter, the bidirectional sequence for *Ri*ZS1 and 4CL-BAS(S331V) was integrated into the GRE3 locus of the previously engineered yeast strain using homologous recombination producing H6712. In the same way, yeast strain H6713 was made utilizing a natural BAS sequence instead of BAS(S331V).

**Table 2:** Vectors and Strains

vectors and strains	description
Vector *N. benthamiana*	
p_Nb4CL-BAS_M	pK2GW7-*Nb*-SES *At*4CL GSG-linker *Rp*BAS(S331V-mutation)
p_Nb4CL-BAS	pK2GW7-Nb*-*SES *At*4CL GSG-linker *Rp*BAS
p_NbBAS	pK2GW7-*Nb*-SES *Rp*BAS
p_Nb4CL	pK2GW7-*Nb*-SES *At*4CL
p_Nb-ZSI	pK2GW7-*Nb*-SES *Ri*ZS1
Vector *S. cerevisiae*	
B10598	pRSET_Sc-SES_*Pc*4CL-Bidir-*Rp*BAS
pR_ScZS1_4CL-BAS_M	pRSET_*Sc*-SES_RiZS1-bidir-*Pc4*CL-GSG-*Rp*BAS(S331V-mutation)
pR_ScZS1_4CL-BAS	pRSET_*Sc*-SES_*Ri*ZS1-Bidir-*Pc4*CL-GSG-*Rp*BAS
pL_TAL_ARO7	pLL-x_X-1in-*Sc*-SES_FjTAL-bidir-ARO7
Strains	
H4590	CEN_PK102–5B + URA3 in_TDH3cP_BM3RI-VP16AD (SES)
H5304	H4590 + 2 BS(Bm3R1)-114cP-ARO4(K229L)
H6707	H4590 + Sp-HIS5-8BS-BID-Pc4CL_RpBAS
H6708	H5304 + Sp-HIS5_8BS-201cPRiZS1-114cP4CL-GSG-BAS
H6709	H5304 + Sp-HIS5_8BS-201cPRiZS1-114cP4CL-GSG-BAS(S331V)
H6710	H5304 + pLEU2in_114cPNpgA-201cPACC1-2xmut
H6711	H6710 + pLL-x_X-1in-PT-BID_201cPFjTAL-114cPARO7
H6712	H6711 + Sp-HIS5_8BS-BID-201cPRiZS1-114cPPc4CL-GSG-BAS(S331V)
H6713	H6711 + Sp-HIS5_8BS-BID-201cPRiZS1-114 cPPc4CL-GSG-BAS

### Extraction and Preparation of Tobacco Samples

*N. benthamiana* leaves were collected 6 days after infiltration and immediately frozen in liquid nitrogen. Samples were lyophilized (CHRIST Alpha 1-4 LD Plus, Martin Christ, Osterode am Harz, Germany) and powdered with a ball mill grinder (MM301, RETSCH, Haan, Germany) for 2 × 1 min at 29 Hz. Lyophilized samples were weighed with a precision scale, and 100 mg of each was suspended in 5 mL of 80% (v/v) aqueous methanol and sonicated for 20 min, followed by centrifugation for 15 min at 3000 rpm. Two methanol extractions were performed for each sample, and supernatants were combined into 50 mL Falcon tubes and evaporated to dryness (SpeedVac SPD 300DDA, Thermo Scientific, Bellefonte, USA).

Crude plant extract samples were mixed with 500 μL of 10 mM (MES)-KOH (2-(*N*-morpholino) ethanesulfonic acid) and 150 μL of 5 mg/mL almond β-glycosidase (Sigma-Aldrich, St. Louis, USA) and incubated at 37 °C for 15 h with gentle shaking. Samples were then transferred into glass KIMAX tubes, and raspberry ketone was extracted with 4 mL of methyl *tert*-butyl ether (MTBE), transferred into 2 mL Agilent vials, and evaporated to dryness under nitrogen flow.

### Extraction of Yeast Samples

Samples of yeast cultivations were collected on seven separate days. From each sample, 1 mL was collected into 2 mL Eppendorf tubes and centrifuged at 13 000 rpm to separate the cell mass. The liquid fraction was transferred to a new 2 mL Eppendorf tube and was stored at −20 °C prior to extraction. Extraction of raspberry ketone was carried out in glass KIMAX tubes with 2 mL of MTBE. Samples were agitated with cross bar magnets for 20 min on a magnetic stirrer and centrifuged for 5 min at 3000 rpm with a KIMAX centrifuge, and the supernatant was transferred into 2 mL Agilent vials and evaporated until dry under nitrogen flow.

### Chemical Analysis of Yeast and Tobacco

The sample extracts in 2 mL glass vials were spiked with internal standard (*trans*-cinnamic acid, Sigma-Aldrich, St. Luis, CA, 6.8 μg/sample) and evaporated into dryness under nitrogen flow. The residues were trimethylsilylated at 70 °C for 1 h after adding 50 μL of pyridin and 50 μL of *N*-trimethylsilyl-*N*-methyl trifluoroacetamide (MSTFA) with 1% trimethylchlorosilane (TMCS) as catalyst (MSTFA+1% TMCS (Thermo scientific, Bellefonte, USA)). For quantification, a 5-point (1.2–36 μg/mL) calibration curve was prepared for raspberry ketone (4-(4-hydroxyphenyl)-2-butanone (Sigma-Aldrich, St. Louis, USA)) and 4-point (1.4–11 μg/mL) calibration curves, for raspberry ketone glucoside (MedChemExpress, New Jersey, USA) and rhododendrol (Tokyo Chemical Industry, Tokyo, Japan).

The analyses were performed on Agilent GC-MS instrumentation (7890A GC + 5975C MSD, Agilent Technologies, Inc., Santa Clara, CA, USA), and the data were collected with MassHunter GC-MS data acquisition mode. The 1 μL injections (Gerstel MPS, Gerstel GmbH&Co.KG, Mühlheim an der Ruhr, Germany) were done in splitless mode at 260 °C. An Agilent DB-5 ms (30 m × 250 μm × 0.25 μm) fused silica capillary column was used for chromatography, and the oven temperature was increased from 50 °C (2 min) to 280 °C (10 min) at a rate of 10 °C/min. The MSD transfer line was at 260 °C, while the source and quadrupole temperatures were 230 and 150 °C, respectively. The data were collected in EI scan mode (70 eV) within a mass range of *m*/*z* 30–800 amu. Identification of the compounds was based on retention and spectral data of reference substances, on library comparisons to the NIST ’08 GC-MS library (National Institute of Standards and Technology, Gaithersburg, MD, USA), and on literature data.

### Statistical Analysis

The difference between the constructs producing raspberry ketone, *Ri*ZS1 expressed together with *At*4CL-*Rp*BAS and *At*4CL-*Rp*BAS alone and of *At*4CL-*Rp*BAS with *At*4CL-*Rp*BAS(S331V), was confirmed with a two-tailed student test using *t*-test function for similar variance (Excel, Microsoft).

### *S. cerevisiae* Tolerance Assay of Raspberry Ketone

The tolerance of *S. cerevisiae* for raspberry ketone was assayed by using a bioscreen analyzer (Bioscreen C MBR automated turbidometric analyzer, Growth Curve Ltd., Turku, Finland). Three different yeast strains, H4590, 4CL-BAS, and the *de novo* S331V raspberry ketone producing strain, were exposed to 0–140 mg/L raspberry ketone without additional *p*-HCA feeding. Yeast cultures were diluted to a starting OD_600_ of 0.05 measured with a spectrophotometer (Ultrospec 2100 pro, Amersham Biosciences, Amersham, United Kingdom) and grown overnight. Samples were pipetted into 100 well Bioscreen honeycomb microtiter plates with 200 μL of yeast culture in each well. Raspberry ketone solutions in 99% ethanol were prepared, and 2 μL of the respective dilution was pipetted into each well with five replicates for each concentration and yeast strain.

## Results

### Accumulation of Raspberry Ketone in *N. benthamiana*

**Figure 2:**
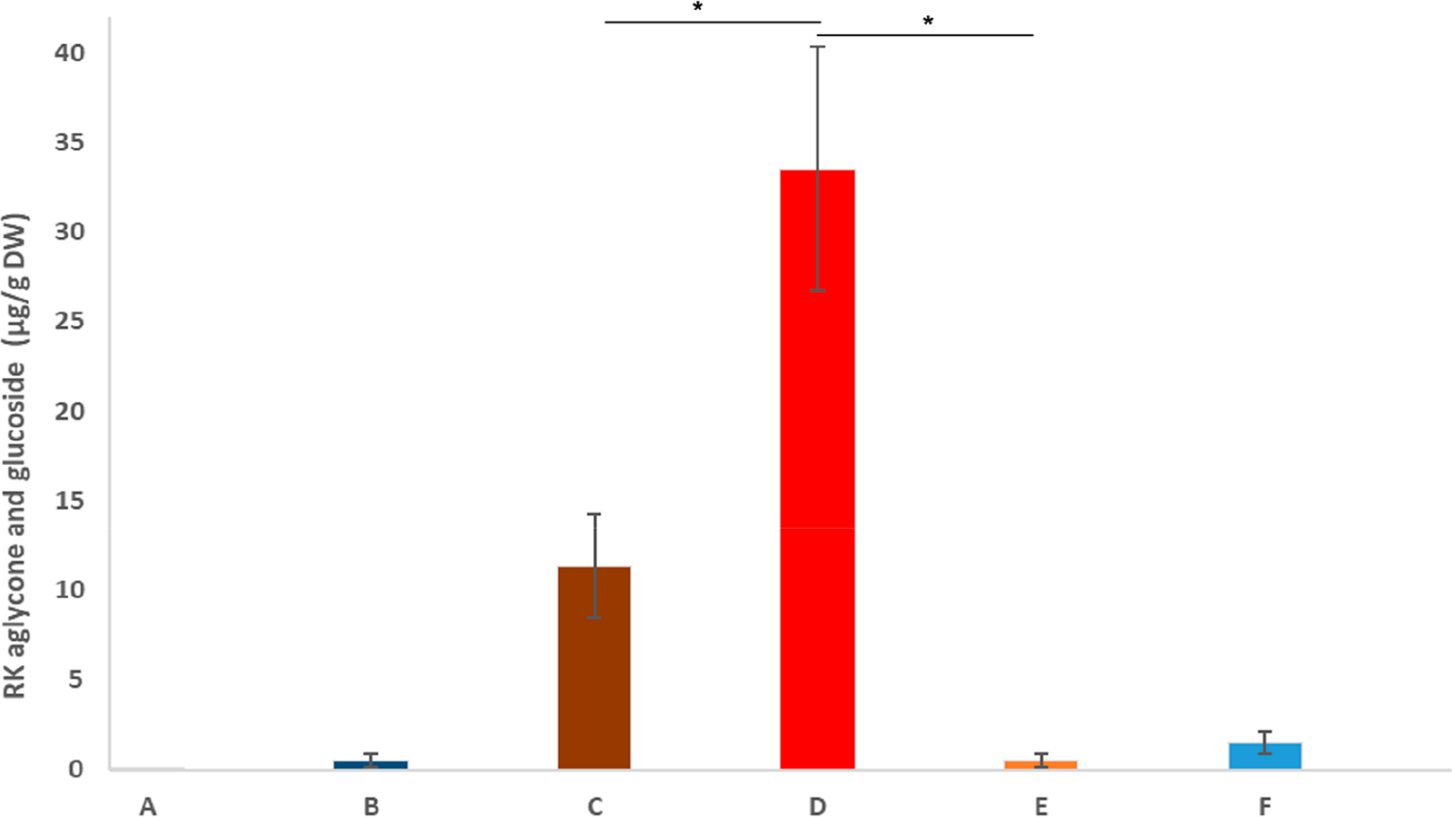
Sum of hydrolyzed raspberry ketone glucosides and aglycone produced transiently in *N. benthamiana* 6 days after infiltration from deglycosylated samples (A–E). (A) *Ri*ZS1 expressed together with *Rp*BAS; (B) *At*4CL expressed together with *Rp*BAS; (C) *Ri*ZS1 expressed together with 4CL-BAS; (D) empty vector expressed together with 4CL-BAS; (E) empty vector expressed together with 4CL-BAS(S331V). (F) Amount of aglycone produced from untreated sample corresponding to (D). Bars represent the mean value ± standard deviation (*n* = 3). *: Significant pairwise difference in the mean (Student’s *t*-test, *p* < 0.01); *p*-values < 0.05 are considered statistically significant.

Initial transient expression experiments in *N. benthamiana* were carried out using the *A. tumefaciens* strain EHA-105 carrying *Rp*BAS and *Ri*ZS1 in separate constructs. However, only trace amounts of raspberry ketone glucoside, i.e., less than 0.01 μg/g of dry weight, were detected in some of the infiltrated plant samples. Expressing *Rp*BAS and *At*4CL in separate constructs (Figure 2) showed a slight increase, producing raspberry ketone glucoside up to 0.2 μg/g (Figure 2, bar A). Utilizing a 1:1 mixture of the fusion construct harboring *At*4CL-BAS(S331V) and *Ri*ZS1 increased the production of raspberry ketone glucoside to 0.9 μg/g of dry weight (Figure 2, bar D). Finally, expressing the fusion sequence *At*4CL-*Rp*BAS utilizing the natural unmutated BAS sequence in a 1:1 mixture with *Ri*ZS1 or the same fusion sequence without *Ri*ZS1 but instead together with an empty vector, 11.3 μg/g (±2.8 μg) (Figure 2, bar B) and 33.5 μg/g (±6.8 μg) (Figure 2, bar C) raspberry ketone glucoside dry weight, respectively, was produced. Small amounts of raspberry ketone aglycone (Figure 2, bar F), 1.5 μg/g (±0.6 μg), were detected in leaves infiltrated with the *At*4CL-BAS fusion sequence. Rhododendrol was also produced transiently in *N. benthamiana*, with *At*4CL-BAS, the sum of rhododendrol glucoside (rhododendrin) and aglycone (rhododendrol) being significantly lower than that of raspberry ketone, at a total of 2.1 μg/g (±0.7 μg) dry weight.

### Production of Raspberry Ketone in *S. cerevisiae* with *p*-HCA Feeding

**Figure 3:**
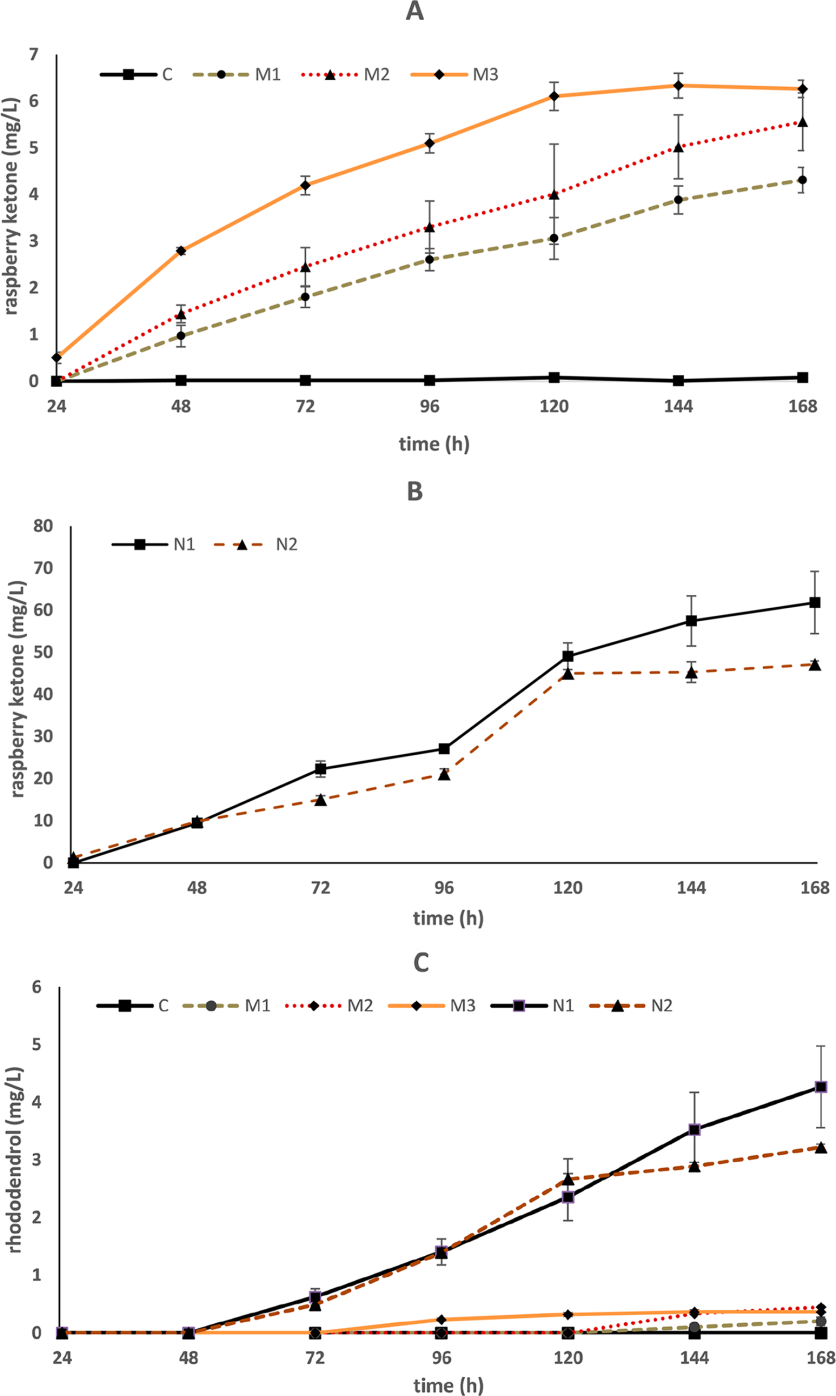
Raspberry ketone (A, B) and rhododendrol (C) production during a seven-day fermentation in five different yeast strains. Yeast strains expressing *Rp*BAS(S331V) mutation: C, control; M1, *Rp*BAS(S331V) expressed under bidirectional promoters together with Pc4CL and *p*-HCA precursor feeding; M2: *Ri*ZS1 and 4CL-BAS(S331V) and *p*-HCA precursor feeding; M3, *de novo* raspberry ketone producing strain harboring *Rp*BAS(S331V). Yeast strains expressing natural BAS: N1, *Rp*BAS fused to Pc4CL expressed with *Ri*ZS1 and *p*-HCA feeding; N2 , *de novo* raspberry ketone producing strain with *Rp*BAS. Bars represent the mean value ± SD (*n* = 3).

The yeast strain expressing Pc4CL and *Rp*BAS(S331V) supplemented with a total of 27 mg of exogenous *p*-HCA as a precursor produced 4.3 mg/L (±0.3 mg) of raspberry ketone after 7 days of fermentation (Figure 3A-M1). The yeast strain incorporating 4CL-BAS(S331V) with *Ri*ZS1 produced a maximum of 5.6 mg/L (±0.6 mg) raspberry ketone when supplemented with a total of 27 mg of *p*-HCA (Figure 3A-M2). Further assessment was conducted to verify whether any difference could be detected between the natural *Rp*BAS and the mutated enzyme. The parental yeast strain H4590 transformed with the same sequence configuration, except for using the natural BAS sequence, led to a significant increase of raspberry ketone production, 61 mg/L (±7 mg) in nonoptimized culture conditions with 27 mg of exogenous *p*-HCA (Figure 3B-N1).

### *De Novo* Biosynthesis of Raspberry Ketone in *S. cerevisiae*

The coumaric acid producing strain, harboring a tyrosine specific gene from *Flavobacterium johnsoniae* (*Fj*TAL)^37^ and overexpressing a feedback-insensitive chorismate mutase *aro*7^G141S^ and feedback-insensitive DAHP synthase *aro*4^K229L^, was expressed with bidirectional sequences of *Ri*ZS1 and 4CL-BAS(S331V) accumulating 6.3 mg/L (±0.2 mg) raspberry ketone (Figure 3A-M3). Subsequently, the strain harboring a natural BAS sequence produced 47 mg/L (±0.8 mg) raspberry ketone, utilizing glucose as carbon source during a seven-day fermentation (Figure 3B-N2). The accumulation of rhododendrol was detected in all raspberry ketone producing strains, ranging from 0.2 to 4.3 mg/L (Figure 3C). Chromatograms of yeast strains producing raspberry ketone clearly showed accumulation of both phloretic acid and phenylethyl alcohol (Figure S1).

### Raspberry Ketone Tolerance Assay for Yeast

**Figure 4:**
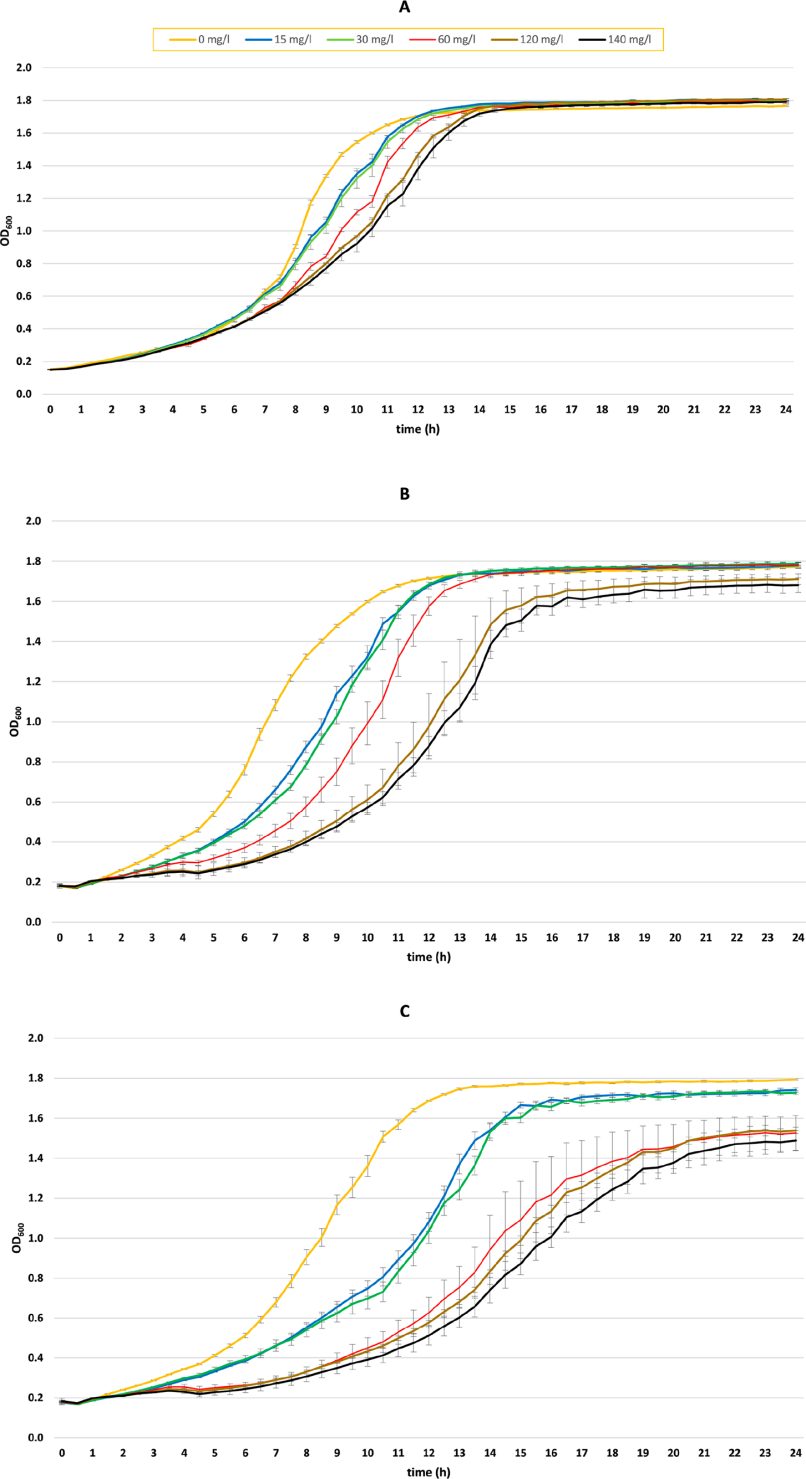
Growth assay for three different yeast strains during a 24 h incubation at 30 °C with externally supplied raspberry ketone without added *p*-HCA. (A) Control strain H4590 containing SES synthetic transcription factor. (B) Yeast strain RiZS1_4Cl-BAS which needs *p*-HCA feeding to produce raspberry ketone. (C) *De novo* raspberry ketone producing strain RiZS1_4CL-BAS(S331V). Bars represent the mean value ± SD (*n* = 3).

The tolerance of *S. cerevisiae* toward raspberry ketone analyzed with a growth assay comparing three yeast strains supplemented with raspberry ketone concentrations of 0–140 mg/L is shown in Figure 4. The assay shows that externally supplied raspberry ketone differentially affects the growth of *S. cerevisiae*. The H4590 control yeast strain reached the stationary growth phase within 12 h and the highest concentration of 140 mg/L raspberry ketone within 14 h (Figure 4A). The RiZS1_4Cl-BAS strain attained stationary phase 13 h after initiation with a similar progressive delay along the increasing concentration until 60 mg/L. With the addition of 120 and 140 mg/L, the stationary phase is reached at around 16 h, however, at a lower OD_600_ level. The *de novo* raspberry ketone-producing yeast strain was noticeably more sensitive for increasing levels of raspberry ketone, barely reaching stationary growth phase at 24 h with 60–140 mg/L raspberry ketone and with a low OD_600_ value of 1.5. Addition of 15 and 30 mg/L raspberry ketone also affected the growth, reaching stationary growth phase at 15 h. The growth curves of the unfed yeasts were similar for all three strains.

## Discussion

In the current study, expressing mutated BAS with BAR, i.e., *Ri*ZS1, proved to be inefficient in producing raspberry ketone transiently in tobacco. Expressing 4CL and BAS was only marginally more efficient. The fusion sequence 4CL-BAS(S331V) improved the production of raspberry ketone but not remarkably. Nonmutated BAS linked to 4CL was finally tested to compare the efficiency of the two enzymes in producing raspberry ketone, which in this case yielded a remarkable increase of up to 40 μg/g of dry weight of raspberry ketone glucoside. It has been noted in microbial fermentation experiments^1,9,26^ and in bioconversion experiments in plants^28^ that raspberry ketone can be produced in the absence of *Ri*ZS1, hence highlighting that many organisms inherently possess enzymes with BAR activity. It was therefore not a surprise that *Ri*ZS1 did not improve the production of raspberry ketone in tobacco, but unexpectedly, the production was even substantially lower when expressed together with 4CL-BAS. This could be explained by the reaction of cosuppression, a phenomenon that has been intensely studied in plants,^38^ in this case of the transgene, and similar endogenous reductase genes.

It is possible that the inefficiency of the mutated BAS is due to the pH dependence of the modified enzyme activity,^16,22^ substrate specificity, or some other structure-related reasons. According to Abe et al.,^22^ the optimal pH range for BAS(S331V) is 7.5 to 8.5 without enzyme activity at pH 6. Natural BAS behaves similarly in the higher optimal range, not showing activity at pH 6.5. Unlike the S331V mutation, enzyme activity is, however, restored in acidic pH values under 6.5. The cytosolic pH of *S. cerevisiae* has been shown to fluctuate between 5.7 and 7, depending on the availability of glucose,^39^ and the cytosolic pH of *Arabidopsis thaliana* has been demonstrated to be slightly alkaline in the range of 7.3.^40^ Various factors influence the cytosolic pH in plants. A drastic drop of 0.5 to 1 pH units was observed in *N. tabacum* and *S. tuberosum*(41) under saline stress. Furthermore, it has been observed that cytoplasmic acidification can be a defense reaction in plants.^42^ The infiltration process, inducing both saline and pathogenic stress, could result in a significant decrease of cytosolic pH, thus preventing the mutated BAS from functioning.

In the current research, we show for the first time that raspberry ketone can be successfully produced transiently in tobacco by utilizing a fusion sequence method.^25,26,43^ The success is most likely due to the channeling of an adequate amount of *p*-coumaroyl-CoA from the CoA ligase to the synthase. When 4CL is linked directly to BAS, the putative interaction of the enzymes could facilitate direct transfer of the CoA to the active site of the synthase, thus negating loss of the intermediate compound to competitive pathways or possibly by diffusion.^43^ Recent work on metabolic engineering toward the production of phenylpropanoids and raspberry ketone in transgenic tobacco suggested that the accumulation of volatile raspberry ketone aglycones is cell specific and is produced in fruits and flowers.^44^ Our research based on transient expression confirms that raspberry ketone glucosides are the main compounds produced in tobacco leaves with only small but measurable amounts of aglycones. Deglycosylated extracts from tobacco leaf samples had a distinct raspberry-like fragrance, and raspberry ketone was confirmed by GC-MS analysis. Flavor enhancement could be a future prospect in cellular agriculture, which has gained interest both from the public and from the industrial sector. Plant cells can be grown in bioreactors and utilized in various ways for food and cosmetics^45,46^ ,but quite often lack flavor and fragrance. Genetic engineering of plants toward producing more flavors could make cellular agriculture more attractive to consumers.

Even though yeast strains engineered with 4CL and BAS either separately or fused together with a linker sequence were capable of producing significant amounts of raspberry ketone aglycone, the process requires exogenous addition of the precursor molecule *p*-HCA. The pathway leading to the production of *p*-HCA can be achieved either via a two-step enzymatic reaction from phenylalanine utilizing PAL and C4H or by a single enzymatic step from tyrosine via TAL. Here, we utilized a highly efficient TAL gene from *F. johnsoniae* reported by Jendresen et al.^37^ for *de novo* production of raspberry ketone. We also performed metabolic engineering of the phenylpropanoid pathway of *S. cerevisiae* by upregulating the genes *ARO4* and *ARO7* with feedback insensitive mutations to channel the pathway toward producing more *p*-HCA. The highest amount of raspberry ketone measured in our nonoptimized production system was over 60 mg/L when fed with the precursor *p*-HCA and utilizing a natural BAS fused to 4CL. *De novo* yeast fermentation produced up to 47 mg/L. Both values mark, to our knowledge, the highest amounts of raspberry ketone ever reported in a yeast system.

Interestingly, raspberry ketone was not the only compound accumulating in the *de novo* producing strains. Rhododendrol, which is a reduced derivative of raspberry ketone, was detected in all of the yeast samples but only in the tobacco samples expressing natural *Rp*BAS fused to *At*4CL in the form of rhododendrin. Our observation of rhododendrol accumulation in *N. benthamiana* is aligned with recent work on stable production of raspberry ketone in *N. tabacum*.^44^ Our work suggests that the reduction of 4-hydroxybenzalacetone to raspberry ketone and similarly the reduction of raspberry ketone^9,28^ to rhododendrol are achieved by nonspecific reductases functioning at least in *N. tabacum*(44) and in human liver microsomes.^47^

Phloretic acid is another unique compound produced by our *de novo* raspberry ketone yeast strains, and the fact that it was not detected in the parental *p*-HCA-producing yeast strain nor the control suggested that *p*-coumaric acid is not a precursor for the compound. It has been however previously shown that excessive amounts of phloretic acid are produced from *p*-coumaroyl-CoA in yeast transformed with 4-coumarate:CoA ligase.^48^ A yeast knock out screening proved that the conversion from *p*-HCA to *p*-coumaroyl-CoA is accomplished via an endogenous enzymatic reaction utilizing an enoyl reductase Tsc13.^49^ Furthermore, complementing endogenous Tsc13 with a homologous gene from plants eliminated the unwanted production of phloretic acid. This approach could be implemented in the further metabolic engineering of raspberry ketone production in yeast to avoid the undesired shunt product.

The production of phenylethyl alcohol,^50^ a compound with a floral aroma that is naturally produced in yeast, was greatly enhanced in *de novo p*-coumaric acid producing yeast strains (Figure S1). It is possible that the Aro4 and Aro7 feedback insensitive mutations affect the shikimate pathway and enhance the formation of phenylalanine, which is a precursor of phenylethyl alcohol.

The production of raspberry ketone plateaued in *de novo* raspberry ketone-producing yeast strains after day four. Therefore, we aimed to analyze the effect of raspberry ketone on the growth of yeast and tolerance toward raspberry ketone. Surprisingly, all three yeast strains tolerated externally added raspberry ketone at high concentration, although there was a clear difference in the growth rate between the control strain and the *de novo* raspberry ketone-producing stain. The differential response of the strains to higher concentration of raspberry ketone could indicate different energetic costs related to the expressed set of genes. Alternatively, this phenomenon could be explained by higher raspberry ketone concentration preventing the secretion of raspberry ketone to the medium. Subsequently, this might trigger levels of growth inhibition for *de novo* strains with already high internal levels of raspberry ketone.

## Conclusion

While using genetically modified organisms is still not a viable option in all countries, utilizing synthetic biology and metabolic engineering offers intriguing possibilities for the prospects of flavor enhancement of beverages and plant cellular agriculture. Ultimately, flavor compounds may be produced in a sustainable way and would also be available for various applications ranging from the food to cosmetic industries.

## Supporting Information Available

Sequences of genes used in the study, either as synthetic genes ordered from the provider or created by PCR (Table S1); GC-MS chromatogram of four yeast samples exhibiting differences in peaks for various compounds (Figure S1) (PDF)
