# Supplementary material for: Raspberry Ketone Accumulation in Nicotiana benthamiana and Saccharomyces cerevisiae by Expression of Fused Pathway Genes
Source: J Agric Food Chem. 2023 Sep 1;71(36):13391–400. doi: 10.1021/acs.jafc.3c02097 (PMC10510385; doi:10.1021/acs.jafc.3c02097)
Supplement: Supplementary file 1 — jf3c02097_si_001.pdf [file jf3c02097_si_001.pdf]

Raspberry ketone accumulation in *Nicotiana benthamiana* and *Saccharomyces cerevisiae* by expression of fused pathway genes

Markus Laurel†, Dominik Mojzita†, Tuulikki Seppänen-Laakso†, Kirsi-Marja Oksman-Caldentey†, Heiko Rischer†*

†VTT Technical Research Centre of Finland Ltd, P.O. Box 1000, FI-02044, Finland
*corresponding author: Phone: +358-20-722-4461; Email: heiko.rischer@vtt.fi

Supporting Information

Table S1

Sequences of genes used in the study, either as synthetic genes ordered from provider, or created by PCR

*Sc_Ri*ZS1 (Synthetic gene)	ACCAATCAACTACCTCTACAACTCCAATACACTTAATTAAAATGGCCTCCGGTGGTGAAATGCAAGTTTCTAACA<br>AACAAGTCATCTTCAGAGATTACGTCACTGGCTTTCCAAAAGAATCCGATATGGAATTGACCACCAGATCCATTA<br>CTTTGAAATTGCCACAAGGTTCTACCGGCTTGTTGTTGAAAAACTTGTACTTGTCTTGCGACCCTTACATGAGAG<br>CTAGAATGACTAATCATCACAGGTTGTCCTACGTTGATTCTTTTAAACCAGGTTCTCCAATCATCGGTTATGGTG<br>TTGCTAGAGTTTTGGAATCTGGTAACCCAAAGTTTAACCCAGGTGATTTGGTTTGGGGTTTTACTGGTTGGGAAG<br>AATACTCTGTTATTACCGCTACTGAGTCCTTGTTCAAGATTCATAATACCGACGTTCCCTTGTCTTACTACACTG<br>GTTTGTTGGGTATGCCAGGTATGACTGCTTATGCTGGTTTTTACGAAATTTGCTCTCCAAAGAAAGGTGAAACCG<br>TTTATGTTTCTGCTGCTTCTGGTGCTGTTGGTCAATTGGTCGGTCAATTTGCTAAGTTGACTGGTTGTTATGTTG<br>TTGGTTCTGCCGGTTCTAAAGAAAAGGTTGATTTGCTGAAGAACAAGTTCGGTTTCGATGAAGCCTTCAACTACA<br>AAGAAGAAGCTGATTTGGACGCTGCTTTGAGAAGATATTTTCCAGATGGTATCGACATCTACTTCGAAAATGTTG<br>GTGGTAAGATGTTGGATGCTGTTTTGCCAAATATGAGGCCAAAAGGTAGAATTGCTGTTTGCGGTATGATTTCCC<br>AGTACAATTTGGAACAACCAGAAGGTGTCAGAAACTTGATGGCTTTGATTGTCAAGCAAGTCAGAATGGAAGGTT<br>TCATGGTTTTCTCTTACTATCACTTGTACGGCAAGTTCTTGGAAACAGTTTTGCCTTACATTAAGCAGGGTAAGA<br>TTACCTACGTCGAAGATGTTGTTGATGGTTTGGATAATGCTCCAGCTGCTTTAATTGGTCTGTACTCTGGTAGAA<br>ACGTTGGTAAGCAAGTTGTTGTTGTGTCCAGAGAATGAGTCGACGCTAATTAACATAAAACTCATGATTCAAC
*Sc_Rp*BAS (Synthetic gene)	ATGGCTACCGAAGAGATGAAGAAATTGGCTACTGTTATGGCTATTGGTACTGCTAATCCACCAAACTGTTATTAC<br>CAAGCTGATTTCCCAGACTTCTACTTCAGAGTTACCAACTCTGATCACCTGATCAACTTGAAGCAAAAGTTCAAG<br>AGATTGTGCGAGAACTCCAGAATTGAGAAGAGATACTTGCATGTCACCGAGGAAATCTTGAAAGAGAATCCAAAT<br>ATTGCTGCTTACGAAGCCACTTCCTTGAACGTTAGACATAAGATGCAAGTTAAGGGTGTTGCCGAATTGGGTAAA<br>GAAGCTGCTTTGAAAGCAATCAAAGAATGGGGTCAACCTAAGTCCAAGATTACCCATTTGATCGTTTGTTGTTTG<br>GCCGGTGTTGATATGCCAGGTGCTGATTATCAATTGACCAAGTTGTTGGATTTGGACCCATCTGTTAAGAGGTTC<br>ATGTTTTACCATTTGGGTTGTTATGCTGGTGGTACTGTTTTGAGATTGGCTAAAGATATTGCCGAGAACAACAAA<br>GGTGCTAGAGTTTTGATTGTCTGCTCTGAAATGACTACCACTTGTTTTAGAGGTCCATCTGAAACTCACTTGGAT<br>TCCATGATTGGTCAAGCCATTTTAGGTGATGGTGCTGCTGCTGTTATAGTTGGTGCTGATCCAGATTTGACTGTT<br>GAAAGACCAATCTTCGAATTGGTTTCTACTGCTCAAACTATCGTTCCAGAATCTCATGGTGCTATTGAAGGTCAT<br>TTGTTGGAATCTGGTTTGTCCTTCCACTTGTACAAAACTGTTCCAACCTTGATCTCCAACAACATTAAGACCTGT<br>TTGTCTGATGCTTTCACCCCATTGAATATCTCTGATTGGAATTCCTTGTTCTGGATTGCTCATCCAGGTGGTCCA<br>GCTATTTTGGATCAAGTTACTGCTAAAGTCGGTTTGGAGAAAGAAAAGTTGAAGGTTACCAGACAAGTCTTGAAG<br>GATTACGGTAATATGTCATCTGCTACCGTGTTCTTCATTATGGACGAAATGCGTAAAAAGTCCTTGGAAAATGGT<br>CAAGCTACTACTGGTGAAGGTTTGGAATGGGGTGTTTTGTTTGGTTTTGGTCCAGGTATTACCGTTGAAACTGTT<br>GTCTTGAGATCCGTTCCAGTTATCTCTTGACTCGAGGGGTCCAGCCAGTAAAATCCATACTCAACG
*Sc_Pc*4CL (PCR fragment)	CCTACACTCTACATATCCACACCAATCTACTACAATTATTAATTAAAATGGGTGACTGTGTTGCTCCAAAGGAAG<br>ATTTGATTTTTAGATCTAAGTTGCCAGATATCTATATCCCAAAGCATTTGCCATTGCATACATACTGTTTCGAAA<br>ACATCTCTAAGGTTGGTGACAAGTCATGTTTGATTAATGGTGCTACTGGTGAAACTTTTACTTACTCTCAAGTTG<br>AATTGTTGTCTAGAAAGGTTGCATCAGGTTTGAATAAGTTAGGTATTCAACAAGGTGACACAATTATGTTGTTGT<br>TGCCAAACTCACCAGAATATTTCTTTGCATTTTTGGGTGCATCTTACAGAGGTGCTATTTCAACAATGGCAAATC<br>CATTTTTCACTTCTGCTGAAGTTATTAAACAATTGAAGGCTTCATTGGCAAAGTTGATCATCACTCAAGCATGTT<br>ACGTTGATAAGGTTAAGGATTACGCTGCAGAAAAGAATATCCAAATCATCTGTATCGATGATGCTCCACAAGATT<br>GTTTACATTTCTCTAAGTTGATGGAAGCAGATGAATCAGAAATGCCAGAAGTTGTTATTGATTCTGATGATGTTG<br>TTGCTTTACCATACTCTTCAGGTACTACAGGTTTGCCAAAGGGTGTTATGTTGACACATAAGGGTTTGGTTACTT

	CAGTTGCACAACAAGTTGATGGTGACAACCCAAATTTGTACATGCATTCTGAAGATGTTATGATCTGTATCTTGC<br>CATTGTTCCATATCTATTCATTGAACGCTGTTTTATGTTGTGGTTTGAGAGCAGGTGTTACAATTTTGATTATGC<br>AAAAATTTGATATTGTTCCATTTTTGGAATTGATCCAAAAGTACAAGGTTACTATTGGTCCATTTGTTCCACCAA<br>TTGTTTTAGCTATCGCAAAGTCTCCAGTTGTTGATAAGTACGATTTGTCATCTGTTAGAACAGTTATGTCAGGTG<br>CTGCACCATTAGGTAAAGAATTGGAAGATGCTGTTAGAGCAAAGTTCCCAAACGCTAAGTTAGGTCAAGGTTATG<br>GTATGACTGAAGCTGGTCCAGTTTTAGCAATGTGTTTGGCTTTCGCAAAGGAACCATACGAAATTAAATCTGGTG<br>CTTGTGGTACAGTTGTTAGAAACGCAGAAATGAAGATCGTTGATCCAGAAACTAATGCTTCATTGCCAAGAAACC<br>AAAGAGGTGAAATCTGTATCAGAGGTGACCAAATCATGAAGGGTTATTTGAACGATCCAGAATCTACAAGAACTA<br>CAATTGATGAAGAAGGTTGGTTGCATACTGGTGACATTGGTTTTATTGATGATGATGATGAATTGTTTATTGTTG<br>ATAGATTGAAGGAAATTATTAAGTACAAAGGTTTTCAAGTTGCTCCAGCAGAATTGGAAGCTTTGTTGTTGACAC<br>ATCCAACTATCTCTGATGCTGCAGTTGTTCCAATGATTGATGAAAAAGCTGGTGAAGTTCCAGTTGCATTCGTTG<br>TTAGAACAAACGGTTTTACTACAACTGAAGAAGAAATTAAACAATTTGTTTCTAAGCAAGTTGTTTTCTATAAGA<br>GAATTTTTAGAGTTTTCTTTGTTGATGCTATCCCAAAGTCTCCATCAGGTAAAATCTTGAGAAAGGATTTGAGAG<br>CTAAAATTGCATCTGGTGACTTGCCAAAAGGATCTGGCATGGCTACAGAAGAAATGAAGAAATTGGCAACAGTTA<br>TGGC
*Sc_Fj*TAL (synthetic gene)	CTACATATCCACACCAATCTACTACAATTAATTTAAATAATGAACACCATCAACGAATACTTGTCCTTGGAAGAA<br>TTCGAAGCCATCATCTTCGGTAATCAAAAGGTTACCATCTCCGATGTTGTTGTCAACAGAGTTAACGAATCCTTC<br>AACTTCTTGAAAGAATTCTCCGGTAACAAGGTTATCTACGGTGTTAATACTGGTTTTGGTCCAATGGCTCAATAC<br>AGAATCAAAGAATCCGACCAAATCCAATTGCAATACAACTTGATCAGATCCCACTCTTCTGGTACTGGTAAACCA<br>TTGTCTCCAGTTTGTGCTAAAGCTGCTATTTTGGCTAGATTGAACACTTTGTCTTTGGGTAATTCAGGTGTTCAC<br>CCATCCGTTATTAACTTGATGTCCGAATTGATCAACAAGGACATTACCCCATTGATCTTTGAACATGGTGGTGTT<br>GGTGCTTCAGGTGATTTGGTTCAATTGTCTCATTTGGCCTTGGTTTTGATTGGTGAAGGTGAAGTTTTTTACAAG<br>GGTGAAAGAAGACCAACCCCAGAAGTTTTTGAAATCGAAGGTTTGAAGCCAATCCAAGTCGAAATTAGAGAAGGT<br>TTGGCTTTGATCAACGGTACTTCTGTTATGACTGGTATCGGTGTTGTTAATGTTTACCACGCTAAGAAGTTGTTG<br>GATTGGTCCTTGAAATCTTCCTGCGCTATTAACGAATTGGTTCAAGCTTACGATGATCACTTCTCCGCTGAATTG<br>AATCAAACTAAGAGACACAAGGGTCAACAAGAAATTGCCTTGAAGATGAGACAAAACTTGTCCGATTCTACCTTG<br>ATTAGAAAGAGAGAAGATCACTTGTACTCCGGTGAAAACACCGAAGAAATCTTCAAAGAAAAGGTCCAAGAATAT<br>TACTCCTTGAGATGCGTTCCACAAATTTTGGGTCCAGTTTTGGAAACCATTAACAATGTTGCCTCCATCTTGGAA<br>GATGAATTCAACTCTGCTAACGACAACCCAATCATCGATGTTAAGAATCAACACGTTTACCATGGTGGTAATTTC<br>CACGGTGATTACATCTCATTGGAAATGGACAAGTTGAAGATCGTCATTACCAAGTTGACTATGTTGGCCGAAAGA<br>CAATTGAATTACTTGTTGAACTCCAAAATCAATGAATTATTGCCACCATTCGTCAATTTGGGTACTTTGGGTTTT<br>AACTTCGGTATGCAAGGTGTTCAATTCACTGCTACTTCTACTACTGCTGAATCCCAAATGTTGTCTAACCCTATG<br>TACGTTCATTCCATCCCAAACAACAACGACAATCAAGACATAGTCTCTATGGGTACAAACTCCGCTGTTATTACC<br>TCCAAGGTTATTGAAAACGCCTTCGAAGTTTTGGCCATTGAAATGATTACTATCGTTCAAGCCATCGACTACTTG<br>GGTCAAAAGGATAAGATTTCCTCCGTCAGTAAAAAGTGGTACGACGAAATCAGAAACATCATCCCAACTTTCAAA<br>GAAGATCAAGTCATGTACCCATTCGTCCAAAAAGTTAAGGATCACTTGATTAACAACTGAGTCGACGCTAATTAA<br>CATAAAACTCATGATTCAACGTTTGTG
Sc_Aro7(G141S) (Synthetic gene)	CCAATCAACTACCTCTACAACTCCAATACACTTAATTAAAATGGATTTCACAAAACCAGAAACTGTTTTAAATCT<br>ACAAAATATTAGAGATGAATTAGTTAGAATGGAGGATTCGATCATCTTCAAATTTATTGAGAGGTCGCATTTCGC<br>CACATGTCCTTCAGTTTATGAGGCAAACCATCCAGGTTTAGAAATTCCGAATTTTAAAGGATCTTTCTTGGATTG<br>GGCTCTTTCAAATCTTGAAATTGCGCATTCTCGCATCAGAAGATTCGAATCACCTGATGAAACTCCCTTCTTTCC<br>TGACAAGATTCAGAAATCATTCTTACCGAGCATTAACTACCCACAAATTTTGGCGCCTTATGCCCCAGAAGTTAA<br>TTACAATGATAAAATAAAAAAAGTTTATATTGAAAAGATTATACCATTAATTTCGAAAAGAGATGGTGATGATAA<br>GAATAACTTCTCATCTGTTGCCACTAGAGATATAGAATGTTTGCAAAGCTTGAGTAGGAGAATCCACTTTGGCAA<br>GTTTGTTGCTGAAGCCAAGTTCCAATCGGATATCCCGCTATACACAAAGCTGATCAAAAGTAAAGATGTCGAGGG<br>GATAATGAAGAATATCACCAATTCTGCCGTTGAAGAAAAGATTCTAGAAAGATTAACTAAGAAGGCTGAAGTCTA<br>TGGTGTGGACCCTACCAACGAGTCAGGTGAAAGAAGGATTACTCCAGAATATTTGGTAAAAATTTATAAGGAAAT<br>TGTTATACCTATCACTAAGGAAGTTGAGGTGGAATACTTGCTAAGAAGGTTGGAAGAGTAAGGATCCGCGAATTT<br>CTTATGATTTATGATTTTTATTATTAAATAAG
*Nb_Rp*BAS (PCR fragment)	ATGGCAACTGAGGAGATGAAGAAATTGGCCACCGTGATGGCCATTGGCACGGCCAACCCTCCGAACTGCTACTAC<br>CAGGCCGACTTTCCCGACTTCTACTTCCGCGTCACCAACAGCGACCACCTCATCAACCTCAAGCAAAAGTTCAAG<br>CGCCTTTGTGAAAACTCAAGGATTGAGAAGCGTTACCTTCATGTGACCGAAGAGATTCTCAAGGAAAACCCAAAC<br>ATTGCTGCCTACGAGGCAACCTCGTTGAATGTAAGACACAAAATGCAAGTGAAAGGAGTTGCAGAGCTTGGGAAA<br>GAGGCTGCCCTCAAGGCCATCAAAGAATGGGGCCAACCCAAGTCCAAGATCACACATCTCATCGTGTGTTGCCTA<br>GCCGGCGTTGACATGCCCGGCGCGGATTATCAACTCACTAAGCTTCTTGACCTTGACCCTTCCGTCAAGCGTTTT<br>ATGTTTTACCACCTAGGATGCTACGCTGGTGGCACTGTCCTTCGCCTTGCAAAGGACATAGCGGAGAACAACAAG<br>GGAGCTCGTGTTCTCATCGTTTGCTCAGAGATGACAACAACTTGTTTTCGTGGGCCATCTGAAACCCATCTGGAC<br>TCCATGATAGGCCAAGCAATATTAGGCGATGGGGCTGCAGCTGTCATAGTTGGCGCAGATCCAGACCTAACCGTT<br>GAGAGGCCCATATTCGAGTTGGTTTCCACAGCCCAGACTATTGTACCCGAATCCCATGGTGCAATTGAGGGCCAC<br>TTGCTTGAATCTGGACTCAGTTTCCATTTGTACAAGACCGTTCCTACACTAATCTCTAACAACATTAAAACTTGC<br>CTTTCTGATGCTTTCACTCCTCTAAACATTAGCGATTGGAACTCTCTTTTCTGGATCGCACACCCTGGTGGTCCT<br>GCCATCCTAGACCAAGTTACTGCTAAGGTTGGTCTTGAAAAGGAGAAACTCAAGGTAACTAGACAAGTGTTGAAG<br>GACTATGGAAACATGTCGAGTGCTACGGTGTTTTTCATCATGGATGAGATGAGGAAGAAGTCACTCGAAAACGGT<br>CAAGCAACCACTGGAGAAGGGCTCGAGTGGGGTGTTTTGTTTGGGTTCGGGCCTGGAATCACCGTTGAAACTGTA<br>GTGCTACGCAGTGTGCCCGTAATTAGCTAGGGATCCTAATAGCTATATATCTTTCTTACATCATTATTG

*Nb_Rp*BAS(S331V) (Synthetic gene)	ATGGCAACTGAGGAGATGAAGAAATTGGCCACCGTGATGGCCATTGGCACGGCCAACCCTCCGAACTGCTACTAC<br>CAGGCCGACTTTCCCGACTTCTACTTCCGCGTCACCAACAGCGACCACCTCATCAACCTCAAGCAAAAGTTCAAG<br>CGCCTTTGTGAAAACTCAAGGATTGAGAAGCGTTACCTTCATGTGACCGAAGAGATTCTCAAGGAAAACCCAAAC<br>ATTGCTGCCTACGAGGCAACCTCGTTGAATGTAAGACACAAAATGCAAGTGAAAGGAGTTGCAGAGCTTGGGAAA<br>GAGGCTGCCCTCAAGGCCATCAAAGAATGGGGCCAACCCAAGTCCAAGATCACACATCTCATCGTGTGTTGCCTA<br>GCCGGCGTTGACATGCCCGGCGCGGATTATCAACTCACTAAGCTTCTTGACCTTGACCCTTCCGTCAAGCGTTTT<br>ATGTTTTACCACCTAGGATGCTACGCTGGTGGCACTGTCCTTCGCCTTGCAAAGGACATAGCGGAGAACAACAAG<br>GGAGCTCGTGTTCTCATCGTTTGCTCAGAGATGACAACAACTTGTTTTCGTGGGCCATCTGAAACCCATCTGGAC<br>TCCATGATAGGCCAAGCAATATTAGGCGATGGGGCTGCAGCTGTCATAGTTGGCGCAGATCCAGACCTAACCGTT<br>GAGAGGCCCATATTCGAGTTGGTTTCCACAGCCCAGACTATTGTACCCGAATCCCATGGTGCAATTGAGGGCCAC<br>TTGCTTGAATCTGGACTCAGTTTCCATTTGTACAAGACCGTTCCTACACTAATCTCTAACAACATTAAAACTTGC<br>CTTTCTGATGCTTTCACTCCTCTAAACATTAGCGATTGGAACTCTCTTTTCTGGATCGCACACCCTGGTGGTCCT<br>GCCATCCTAGACCAAGTTACTGCTAAGGTTGGTCTTGAAAAGGAGAAACTCAAGGTAACTAGACAAGTGTTGAAG<br>GACTATGGAAACATGGTGAGTGCTACGGTGTTTTTCATCATGGATGAGATGAGGAAGAAGTCACTCGAAAACGGT<br>CAAGCAACCACTGGAGAAGGGCTCGAGTGGGGTGTTTTGTTTGGGTTCGGGCCTGGAATCACCGTTGAAACTGTA<br>GTGCTACGCAGTGTGCCCGTAATTAGCTAGGGATCCTAATAGCTATATATCTTTCTTACATCATTATTG
*Nb_At*4CL-link (Synthetic gene)	CATAAATCTTCTCAGATCTCTTCCAATTTTCTTTAATTAAAATGGAGAAACAGAGCAACAACAACAACAGTGACG<br>TCATTTTCCGATCAAAGTTACCGGATATTTACATCCCGAACCACCTATCTCTCCACGACTACATCTTCCAAAACA<br>TCTCCGAATTTGCCACTAAGCCTTGCCTAATCAACGGACCAACCGGCCACGTGTACACTTACTCCGACGTCCACG<br>TCATCTCCCGCCAAATCGCCGCCAATTTTCACAAACTCGGCGTTAACCAAAACGACGTCGTCATGCTCCTCCTCC<br>CAAACTGTCCCGAATTTGTCCTCTCTTTCCTCGCCGCCTCCTTCCGCGGCGCAACCGCCACCGCCGCAAACCCTT<br>TCTTCACTCCGGCGGAGATAGCTAAACAAGCCAAAGCCTCCAACACCAAACTCATAATCACCGAAGCTCGTTACG<br>TCGACAAAATCAAACCACTTCAAAACGACGACGGAGTAGTCATCGTCTGCATCGACGACAACGAATCCGTGCCAA<br>TCCCTGAAGGCTGCCTCCGCTTCACCGAGTTGACTCAGTCGACAACCGAGGCATCAGAAGTCATCGACTCGGTGG<br>AGATTTCACCGGACGACGTGGTGGCACTACCTTACTCCTCTGGCACGACGGGATTACCAAAAGGAGTGATGCTGA<br>CTCACAAGGGTCTAGTCACGAGCGTTGCTCAGCAAGTCGACGGCGAGAACCCGAATCTTTATTTCCACAGCGATG<br>ACGTCATACTCTGTGTTTTGCCCATGTTTCATATCTACGCTTTGAACTCGATCATGTTGTGTGGTCTTAGAGTTG<br>GTGCGGCGATTCTGATAATGCCGAAGTTTGAGATCAATCTGCTATTGGAGCTGATCCAGAGGTGTAAAGTGACGG<br>TGGCTCCGATGGTTCCGCCGATTGTGTTGGCCATTGCGAAGTCTTCGGAGACGGAGAAGTATGATTTGAGCTCGA<br>TAAGAGTGGTGAAATCTGGTGCTGCTCCTCTTGGTAAAGAACTTGAAGATGCCGTTAATGCCAAGTTTCCTAATG<br>CCAAACTCGGTCAGGGATACGGAATGACGGAAGCAGGTCCAGTGCTAGCAATGTCGTTAGGTTTTGCAAAGGAAC<br>CTTTTCCGGTTAAGTCAGGAGCTTGTGGTACTGTTGTAAGAAATGCTGAGATGAAAATAGTTGATCCAGACACCG<br>GAGATTCTCTTTCGAGGAATCAACCCGGTGAGATTTGTATTCGTGGTCACCAGATCATGAAAGGTTACCTCAACA<br>ATCCGGCAGCTACAGCAGAGACCATTGATAAAGACGGTTGGCTTCATACTGGAGATATTGGATTGATCGATGACG<br>ATGACGAGCTTTTCATCGTTGATCGATTGAAAGAACTTATCAAGTATAAAGGTTTTCAGGTAGCTCCGGCTGAGC<br>TAGAGGCTTTGCTCATCGGTCATCCTGACATTACTGATGTTGCTGTTGTCGCAATGAAAGAAGAAGCAGCTGGTG<br>AAGTTCCTGTTGCATTTGTGGTGAAATCGAAGGATTCGGAGTTATCAGAAGATGATGTGAAGCAATTCGTGTCGA<br>AACAGGTTGTGTTTTACAAGAGAATCAACAAAGTGTTCTTCACTGAATCCATTCCTAAAGCTCCATCAGGGAAGA<br>TATTGAGGAAAGATCTGAGGGCAAAACTAGCAAATGGATTGGGATCTGGCATGGCAACTGAGGAGATGAAGAAAT<br>TGGCCACC

ATG: Gibson assembly flanks
GGA: GSG linker sequence
TTA: Restriction enzyme site
GTG: Mutation site

Figure S1

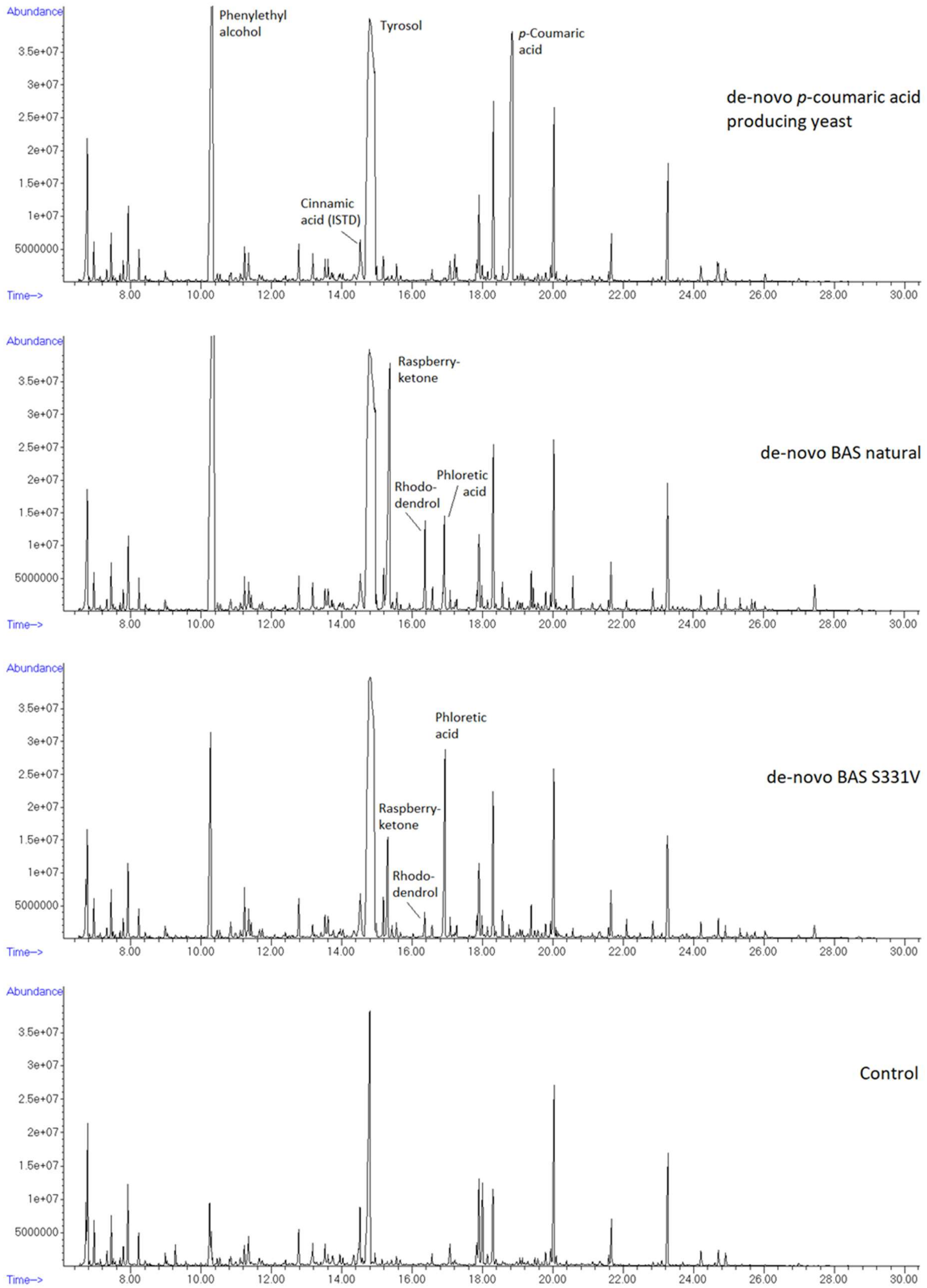

GC-MS chromatogram of four yeast samples exhibiting differences in peaks for various compounds. The yeast strain expressing a natural BAS shows increased levels of rhododendrol and raspberry ketone, whilst the mutated (S331V) strain shows increased levels of phloretic acid. The

chromatogram for de-novo *p*-coumaric acid producing yeast strain does not exhibit peaks for other compounds than *p*-coumaric acid
